# Modulation of Spatial Memory Deficit and Hyperactivity in Dopamine Transporter Knockout Rats *via* α2A-Adrenoceptors

**DOI:** 10.3389/fpsyt.2022.851296

**Published:** 2022-03-25

**Authors:** Natalia Kurzina, Anastasia Belskaya, Arina Gromova, Alla Ignashchenkova, Raul R. Gainetdinov, Anna Volnova

**Affiliations:** ^1^Institute of Translational Biomedicine, Saint Petersburg State University, Saint Petersburg, Russia; ^2^Biological Faculty, Saint Petersburg State University, Saint Petersburg, Russia; ^3^Saint Petersburg University Hospital, Saint Petersburg, Russia

**Keywords:** dopamine transporter knockout (DAT-KO) rats, ADHD model, norepinephrine (NE), dopamine (DA), α2A-adrenoceptors, Guanfacine (GF), Yohimbine (YOH), spatial working memory

## Abstract

Attention deficit hyperactivity disorder (ADHD) is manifested by a specific set of behavioral deficits such as hyperactivity, impulsivity, and inattention. The dopamine neurotransmitter system is postulated to be involved in the pathogenesis of ADHD. Guanfacine, a selective α2A-adrenoceptor agonist, is prescribed for ADHD treatment. ADHD also is known to be associated with impairment of multiple aspects of cognition, including spatial memory, however, it remains unclear how modulation of the norepinephrine system can affect these deficits. Hyperdopaminergic dopamine transporter knockout (DAT-KO) rats are a valuable model for investigating ADHD. The DAT-KO rats are hyperactive and deficient in spatial working memory. This work aimed to evaluate the effects of noradrenergic drugs on the fulfillment of spatial cognitive tasks by DAT-KO rats. The rats were tested in the Hebb – Williams maze during training and following noradrenergic drugs administration. The efficiency of spatial orientation was assessed as to how fast the animal finds an optimal way to the goal box. Testing in a new maze configuration allowed us to evaluate the effects of drug administration after the acquisition of the task rules. The behavioral variables such as the distance traveled, the time to reach the goal box, and the time spent in the error zones were analyzed. It has been observed that α2A-adrenoceptor agonist Guanfacine (0.25 mg/kg) had only a minimal inhibitory effect on hyperactivity of DAT-KO rats in the maze but significantly ameliorated their perseverative pattern of activity and reduced the time spent in the error zones. In contrast, α2A-adrenoceptor antagonist Yohimbine, at the dose of 1 mg/kg, increased the distance traveled by DAT-KO rats and elevated the number of perseverative reactions and the time spent in the error zones. Guanfacine caused minimal effects in wild-type rats, while Yohimbine altered several parameters reflecting a detrimental effect on the performance in the maze. These data indicate that modulation of α2A-adrenoceptor activity potently affects both dopamine-dependent hyperactivity and cognitive dysfunctions. Similar mechanisms may be involved in the beneficial effects of Guanfacine on cognitive deficits in ADHD patients. This study further supports the translational potential of DAT-KO rats for testing new pharmacological drugs.

## Introduction

Attention deficit hyperactivity disorder (ADHD), a neurodevelopmental mental disorder, is characterized by inattention, hyperactivity, and impulsive behavior (1, 2). The ADHD arises in childhood and may persist in adulthood (3, 4). The abnormalities in memory processes were also found in patients with ADHD (5–11). The disruption of spatial working memory in ADHD cases and different approaches to reveal these abnormalities were described in several investigations, including studies performed with the use of a virtual Hebb-Williams maze. In some studies, deficits of attention and spatial working memory are discussed together because they are difficult to separate (12–14). Several hypotheses explain mechanisms underlying this disorder that have not yet been fully validated. However, the key role of abnormalities in the dopamine and the norepinephrine systems in ADHD development was proposed (15, 16).

The dopamine (DA) system is involved in complex behaviors such as decision making (17), motivation (18, 19), reward evaluation (20), social behavior (21), and goal-directed behavior (22, 23). Among several genes associated with the pathogenesis of ADHD, the most noticeable is the gene encoding the dopamine transporter (DAT) (24, 25). It is assumed that norepinephrine (NE) also plays an important role in the pathophysiology of ADHD (26–28). Recent investigations indicated the role of norepinephrine transporter (NET) in ADHD development (29). The NE system is involved in attention processes related to prefrontal cortex (PFC) functions. The PFC receives inputs both from noradrenergic and dopaminergic systems (30–32) and interaction between them seem to be critical for alterations of the PFC functions observed in ADHD (31, 33, 34). It is suggested that the DA role is mainly associated with reward expectancy, whereas NE is involved in the evaluation of the goal and rules of the task (35). Thus, both DA and NE are crucial for PFC cognitive function and probably cannot act independently of each other.

Animal model studies can be helpful for understanding the ADHD mechanisms and elaboration of new diagnostic and therapeutic strategies (22, 36–38). These rodent studies revealed that DA and NE play critical roles in working memory, top-down control, and cognitive flexibility. Low and medium levels of NE and DA in the PFC were found to improve cognitive functions, whereas high concentrations of catecholamines in the PFC negatively affected its functions, indicating that the optimal levels of NE and DA in the PFC are necessary for its proper functioning (39). The blockade of α2A-adrenoceptors in the PFC leads to the ADHD–like behaviors in rodents, including heightened impulsivity and locomotor hyperactivity. In contrast, stimulation of alpha-2-adrenoceptors in the PFC ameliorates these behavioral abnormalities. Thus, effective ADHD treatments should aim at facilitating and optimizing catecholamine transmission in the PFC (40).

The strain of rats with deletion of the DAT gene (DAT knockout rats, DAT-KO rats) was developed in order to investigate the cognitive abnormalities arising from dopamine system dysfunction (37). DAT-KO rats have no dopamine re-uptake and thus have spontaneously elevated extracellular dopamine levels. As a result, they demonstrate novelty-driven hyperactivity and increased stereotypy as well as impaired sensorimotor gating, decreased Y-maze spontaneous alternation, and poor performance of operant tasks (37, 41). Furthermore, they demonstrate deficient spatial working memory and perseverations in various tasks (37, 38, 41–43). Numerous investigations described the dependence of spatial tasks solution on DA transmission (35, 44–46). Accordingly, spatial task fulfillment was impaired in DAT-KO rats (43).

Some drugs prescribed for ADHD treatment in patients affect the NE system. Guanfacine (GF), an α2A-adrenoceptor agonist, was approved by FDA for ADHD treatment in 2009. It directly stimulates postsynaptic α2A-adrenoceptor in the central nervous system and thereby enhances NE neurotransmission (47, 48). The α2A-adrenoceptors are most densely represented in the PFC (30, 49). Guanfacine acts directly on postsynaptic alpha-2A adrenoceptors in the PFC and has beneficial effects on regulating PFC functions (50, 51). The blockade of these receptors by α2-adrenoceptor antagonist Yohimbine (YOH) in the PFC impairs working memory, weakens go-no-go responding, increases locomotor activity in animals. YOH is also used to investigate psychomotor task performance and probe working memory in humans (52–56).

Modulation of DAT-KO rat cognitive processes might result from tenable interrelationships between the noradrenergic and the dopaminergic systems. Several brain areas are critical for complex behaviors in which the noradrenergic and dopamine systems interact closely (16, 51). However, the major interactions between these systems occur in the cortical areas that receive dense NE innervation from the L (57, 58) and dopamine projections from the Ventral Tegmental Area and are responsible for memory and attention (32, 35).

This study aimed to evaluate the effects of α2A-adrenergic drugs on the fulfillment of spatial cognitive tasks in DAT-KO rats.

## Materials and Methods

### Animals

In 10 DAT-KO and 10 control wild-type (WT) rats, males of the same age (3–4 months), were used in the experiments. All experimental procedures were conducted in compliance with The Regulations on Research Using Experimental Animals (Order of Ministry of Health #742), FELASA and Rus-LASA requirements regarding the care and treatment of laboratory animals, and the Ethics committee of Saint Petersburg State University, St. Petersburg, Russia No. 131-03-4 of 24 September 2018.

Before the experiments, rats were maintained in IVC cages (RAIR IsoSystem World Cage 500; Lab Products, Inc.) with free access to food and water, at a temperature of 22 ± 1°C, 50–70% relative humidity and a 12 h light/dark cycle (light from 9 am). Experiments were carried out between 2 pm and 6 pm. For 5 days before the training, rats received food at a ratio of 90% of their regular diet (BioPro, Russia). Each animal was weighed daily prior to the experiment during all the experiments’ duration. At the start of the experiments, the bodyweight of DAT-KO rats was 234.8 ± 1.6 g and was lower than that of WT rats (313.4 ± 2.2 g). At the end of the experiments, the bodyweight of DAT-KO rats was 224.3 ± 1.9 g, and in WT rats, body weight was 269.7 ± 4.8 g.

### Apparatus

**FIGURE 1:**
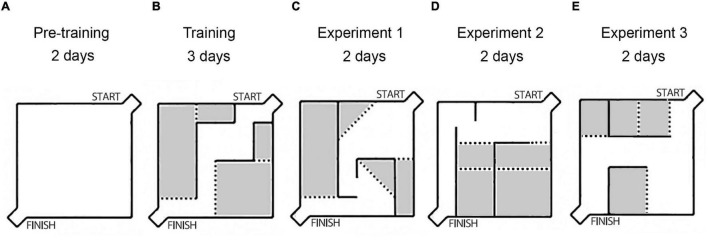
The scheme of the experiments: layouts of the Hebb-Williams maze arenas used in experiments. **(A)** The wall-less maze (pre-training period). **(B)** The unchanging walls arena, training period, without any injections. **(C)** The arena after saline injections. **(D)** The arena after Guanfacine (GF) injections. **(E)** The arena after Yohimbine (YOH) injections. The gray color indicates the error zones of the maze.

We used the Hebb-Williams maze to study animal spatial working memory (59, 60). The Hebb – Williams maze consists of a square area, 75 × 75 cm, with 25 cm walls. One corner is designated as the start of the maze, and the opposing corner contains a goal box with a food well for reward (Figure 1). After testing each animal, the maze surfaces were rubbed with peroxide solution to prevent odor influence.

The behavioral task consisted of searching the path from start to finish to obtain food reinforcement. The interior walls of the maze are moveable, which allows the creation of new arena layouts and different routes through the maze (Figures 1B–E). Different arena layouts were used to diminish habituation’s influence on learning behavioral task rules. Each new arena was coupled with saline or drug injections.

### Task Procedure

#### Pre-training and Training Periods

The pre-training period included a familiarization procedure in a wall-less maze during the first 2 days (Figure 1A), 10 pieces of popcorn breakfast loops (produced by Nestle S.A.) were spread out on the floor surface of the maze, and rats were allowed to individually explore the maze during 10 min. Then rats were trained in the unchanging maze arena (Figure 1B) during 3 days (three trials for each animal per day) with reward only in a goal box food well for habituation to the maze and task rules acquisition.

#### The Experimental Design

Experiment 1 – All rats received saline injections (0.9% NaCl, i.p.), 30 min before each testing and were trained in the new arena configuration for 2 days (Figure 1C). After it the following drugs were used: Experiment 2 (Figure 1D) – Guanfacine (GF; 0.25 mg/kg, i.p., 60 min before testing, Sigma, United States), and Experiment 3 (Figure 1E) – Yohimbine (YOH; 1 mg/kg, i.p., 10 min before testing, Sigma, United States). Each drug was used for two consecutive days. During drug administration the maze configuration was modified every 2 days. Each drug application followed a 2-day drug-free interval.

The behavioral variables such as the distance traveled, the time to reach the goal box, the number of entries into the error zones, time spent in the error zones and number of return were measured and analyzed by a video tracking system (EthoVision XT, Noldus Information Technology, VA) with the video camera being placed above the maze.

### Statistical Analysis

All values were averaged over all trials for 2 days per animal, and then groups of rats were compared. The data were presented as mean ± SEM; *p* < 0.05 was considered statistically significant for all tests. Preliminary estimation of the data distribution normality (Gaussian distribution) was performed using the Kolmogorov–Smirnov test. The paired Mann-Whitney tests and one-way Kruskal–Wallis test combined with Dunn’s multiple-comparison *post hoc* test were used for statistical analysis.

## Results

**FIGURE 2:**
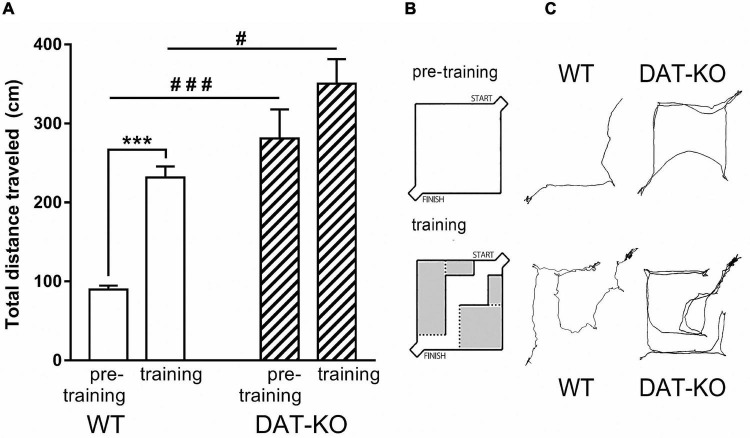
The distances traveled during pre-training and training periods in WT and DAT-KO rats. **(A)** The distances traveled during 30c, ^#^*p* < 0.05; ^###^*p* < 0.001; one-way Kruskal–Wallis test combined with Dunn’s multiple-comparison *post hoc* test; ***p < 0.001; Mann Whitney paired test. **(B)** The wall-less arena in pre-training period and arena in training period. **(C)** Comparison of visual tracking samples; WT, wild-type; DAT-KO, dopamine transporter knockout rats.

The spontaneous hyperactivity of DAT-KO rats was clearly revealed during the pre-training period, when their activity was significantly higher than in WT rats (Figure 2). The distance traveled (Figure 2A) was significantly higher in DAT-KO rats in comparison to WT rats (280.9 ± 36.9 (DAT-KO) vs. 89.5 ± 5.1 (WT), *p* < 0.001; one-way Kruskal–Wallis test combined with Dunn’s multiple-comparison *post hoc* test).

We compared behavioral parameters of DAT-KO and WT rats during pre-training and training periods (Figure 2A). In both cases DAT-KO rats traveled significantly longer distances than WT rats. The pre-training in the wall-less arena reflects the habituation to the new environment, while the arena with internal walls allows investigation of the spatial memory and the acquisition of the task rules. WT rats probably try to remember the optimal route to the finish box and carefully investigate the arena hence their motor activity and distance traveling increase, while hyperactive DAT-KO rats are not able to do it due to their inattentiveness and decreased spatial memory (Figures 2B,C).

Following training (Figure 2A), distances traveled was significantly increased in WT rats (from 89.5 ± 5.1 to 231.5 ± 13.8; *p* < 0.001; Mann Whitney paired test) up to levels observed in untrained DAT-KO rats. In contrast, no significant increase was found during training in DAT-KO rats (280.9 ± 36.0 vs. 350.4 ± 31.0). It should be noted that a substantial change of the internal context of the maze has markedly modified the rat’s behavior. These changes may likely be connected with an interaction of two processes – habituation and learning.

Furthermore, in three experimental sessions (Experiment 1 – vehicle administration; Experiment 2 – GF, 0.25 mg/kg administration, and Experiment 3 – YOH, 1 mg/kg administration), we compared behavioral parameters of DAT-KO and WT rats in the Hebb-Williams maze. The data obtained were compared between two groups of rats and two drugs used.

**FIGURE 3:**
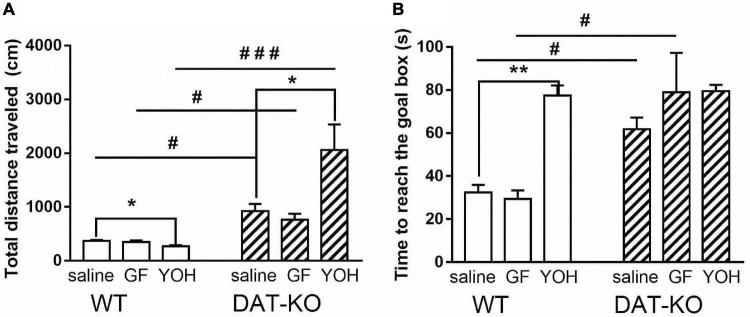
Comparison of distances traveled **(A)** and time to reach the goal box **(B)** by DAT-KO and WT rats during three experimental sessions; ^#^*p* < 0.05; ^###^*p* < 0.001; one-way Kruskal–Wallis test combined with Dunn’s multiple-comparison *post hoc* test; *p < 0.05; **p < 0.01; Mann-Whitney test; WT, wild-type; DAT-KO, dopamine transporter knockout rats.

During all experimental sessions, DAT-KO rats traveled significantly longer distances (Figure 3A) in comparison with WT rats irrespective of the drugs administered (924.1 ± 134.8 vs. 367.1 ± 21.6 for saline, *p* < 0.05; 763 ± 112 vs. 351.9 ± 26.9 for GF, *p* < 0.05; 2062.1 ± 472.2 vs. 268.8 ± 21.2 for YOH, *p* < 0.001; one-way Kruskal–Wallis test combined with Dunn’s multiple-comparison *post hoc* test). The DAT-KO rats showed no differences in distances traveled after GF compared to saline administration, whereas YOH administration increased distances covered (924.1 ± 134.8 (saline) vs. 2062.1 ± 472.2 (YOH); *p* < 0.05; Mann Whitney paired test). After GF injections, the distances traveled by WT rats, just as in DAT-KO rats, did not differ compared to saline injections but slightly decreased after YOH administration (367.1 ± 21.6 (saline) vs. 268.8 ± 21.2 (YOH); *p* < 0.05; Mann Whitney paired test).

The time to reach the goal box (Figure 3B) also significantly increased (*p* < 0.05; one-way Kruskal–Wallis test combined with Dunn’s multiple-comparison *post hoc* test) in DAT-KO rats in comparison with their WT counterparts both after saline (61.8 ± 5.4 vs. 32.5 ± 3.5; *p* = 0.034) and GF (78.9 ± 18.3 vs. 29.5 ± 3.9; *p* = 0.01) injections, and did not differ after YOH administration (Figure 3B). In both groups of rats, no differences in task fulfillment duration after rats’ GF administrations were found. YOH did not significantly change the time to reach the goal box in DAT-KO rats, although it significantly increased this parameter in WT rats (77.5 ± 4.7) compared to vehicle controls (32.5 ± 3.5; *p* < 0.001, Mann Whitney paired test).

**FIGURE 4:**
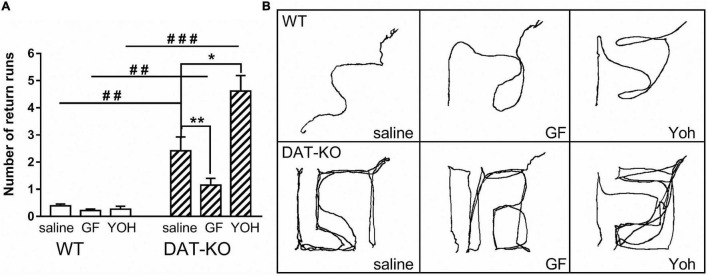
Comparison of the number of return runs reflecting stereotypical (perseverative) patterns of activity **(A)** and examples of movement patterns **(B)** in WT and DAT-KO rats in Experiment 1 (saline injections), Experiment 2 (GF injections), and Experiment 3 (YOH injections); ^###^*p* < 0.001; ^##^*p* < 0.01; one-way Kruskal–Wallis test combined with Dunn’s multiple-comparison *post hoc* test; *p < 0.05; **p < 0.01; Mann Whitney paired test; WT, wild-type; DAT-KO, dopamine transporter knockout rats.

One of the core features of hyperactive DAT-KO animals is the perseverative (stereotypical) pattern of locomotor activity in locomotor activity boxes and increased level of perseverative errors in mazes assessing cognitive performances (43, 61). The analysis of the number of return runs showed (Figure 4A) that the perseverative activity is significantly higher in DAT-KO rats than in WT rats (*p* < 0.001; one-way Kruskal–Wallis test combined with Dunn’s multiple-comparison *post hoc* test). Comparison of stereotypical activity in DAT-KO rats after drugs administration showed that GF injections significantly decreased the occurrence of the perseverative reactions [2.4 ± 0.5 (saline) vs. 1.2 ± 0.03 (GF); *p* = 0.003; Mann Whitney paired test], whereas YOH injections markedly increased perseverative activity [2.4 ± 0.5 (saline) vs. 4.6 ± 0.6 (YOH); *p* < 0.05; Mann Whitney paired test]. WT rats showed a very low level of perseverative activity (Figure 4A), and no drugs affected it.

The perseverative patterns of locomotor activity of DAT-KO animals are also evident when comparing video tracks of the maze exploration during reinforcement searching (Figure 4B). The wild-type animals chose an optimal path to the goal box, while the knockout rats made numerous returns to the start zone, also repeatedly visited the error zones.

**FIGURE 5:**
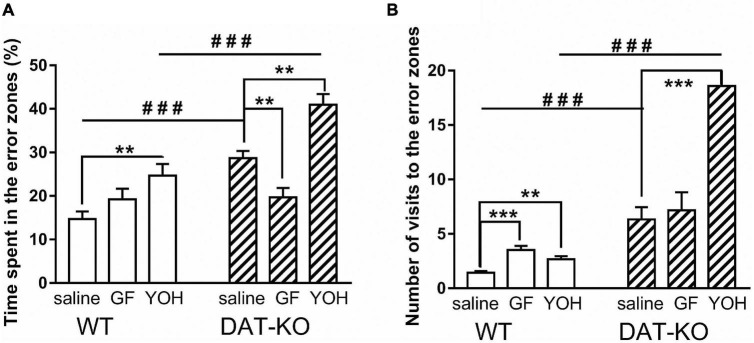
Comparison of the time spent in the error zones of the maze **(A)** and number of visits to the error zones **(B)** of WT and DAT-KO rats; ^###^*p* < 0.001; one-way Kruskal–Wallis test combined with Dunn’s multiple-comparison *post hoc* test; **p < 0.01; ***p < 0.001; Mann Whitney paired test; WT, wild-type; DAT-KO, dopamine transporter knockout rats.

As described previously (43), the DAT-KO rats have deficient spatial memory. In the present study, spatial navigation in DAT-KO rats was less efficient than in WT rats. Saline-treated DAT-KO rats spent more time in error zones [28.7 ± 1.7 (DAT-KO) vs. 14.7 ± 1.8 (WT); *p* < 0.001; one-way Kruskal–Wallis test combined with Dunn’s multiple-comparison *post hoc* test] and visited them more often [6.1 ± 1.1 (DAT-KO) vs. 1.4 ± 0.2 (WT); *p* = 0.0004; one-way Kruskal–Wallis test combined with Dunn’s multiple-comparison *post hoc* test] in comparison to WT rats (Figures 5A,B). The decrease in the time spent in the error zones after GF administration reflects the improvement of task fulfillment by DAT-KO rats [19.7 ± 2.2 (GF) vs. 28.7 ± 1.7 (saline); *p* = 0.003; Mann Whitney paired test] (Figure 5A). In contrast, the injection of YOH produced a significant increase of this parameter [40.9 ± 2.5 (YOH) vs. 28.7 ± 1.7 (saline); *p* = 0.016; Mann-Whitney paired test]. It was found (Figure 5A) that after YOH injections, DAT-KO rats spent a significantly longer time in the error zone than WT rats [40.9 ± 2.5 (DAT-KO) vs. 24.7 ± 2.6; *p* = 0.0005; one-way Kruskal–Wallis test combined with Dunn’s multiple-comparison *post hoc* test]. Both drugs tested in WT rats led to an increase in the time spent in the error zones, but only after YOH they remained in these zones significantly longer (19.2 ± 2.4) in comparison with vehicle conditions (14.7 ± 1.8; *p* = 0.008; Mann Whitney paired test). It should be noted that time spent in the error zones by WT rats was less than in DAT-KO rats after saline and YOH injections.

Similar results were obtained following analysis of the number of error zone visits (Figure 5B). The number of visits to the error zones by WT rats also was significantly lower than in DAT-KO rats for saline injection [1.4 ± 0.2 (WT) vs. 6.3 ± 1.1 (DAT-KO); *p* = 0.0002; one-way Kruskal–Wallis test combined with Dunn’s multiple-comparison *post hoc* test] and for YOH injection [2.7 ± 0.3 (WT) vs. 18.6 ± 2.3; *p* = 0.0006; one-way Kruskal–Wallis test combined with Dunn’s multiple-comparison *post hoc* test]. Interesting, that the injection of YOH produced a significant increase the number of error zone visits as in WT [1.4 ± 0.2 (saline) vs. 2.7 ± 0.3 (YOH); *p* = 0.057; Mann Whitney paired test], as in DAT-KO rats [6.3 ± 1.1 (saline) vs. 18.6 ± 2.3; *p* < 0.001; Mann Whitney paired test] (Figure 5B).

## Discussion

This study evaluated the effects of noradrenergic drugs on DAT-KO rats’ cognitive functions during spatial memory task performance. DAT plays a critical role in regulating both the intraneuronal and extracellular DA homeostasis by controlling the DA re-uptake (62). DAT-KO rats with the deleted DAT gene demonstrate elevated extracellular dopamine levels, spontaneous hyperactivity, perseverations, and cognitive deficits (37). Numerous investigations have documented hyperactivity, abnormal impulsivity, and inattentiveness in DAT-KO rats in various experimental settings (38, 41, 42, 63). Furthermore, our previous studies showed that DAT-KO rats, when performing spatial tasks in the 8-arm radial maze, had a high level of perseverative errors and fulfilled tasks less effectively than their WT counterparts indicative of deficit in working memory (43). We also found that DAT-KO rats can learn non-spatial object recognition tasks under the conditions of novel object presentations and can store this motor task in memory for at least 3 months. Thus, they have a deficiency in learning the cognitive task but can keep in memory the learned task for 3 months and fulfill it even better than during training (64). These data indicate that disruption of the dopamine re-uptake affects cognitive task learning in DAT-KO rats, and proves their translational value to investigate memory and attention deficits related to ADHD.

The role of DA and NE in terms of their differential participation in memory processes is well described. The role of DA in the modulation of the PFC functions was demonstrated in multiple studies in various animal species and humans. It is believed that the dopamine system participates in memory processes through its involvement in reward evaluation. At the same time, the Locus Coeruleus noradrenergic system may exert modulatory effects on the PFC functions through its role in task rule acquisition (35). The DA and NE levels were found to be phasically increased in the rat PFC during cognitive task performance (65). It is speculated that convergence of the DA and NE signaling pathways might have parallel neurophysiological effects. There are data that extracellular DA in the cortex is co-released with NE from noradrenergic terminals, and this co-release is mainly controlled by the α2A-adrenoceptors (66, 67). The α2A-adrenoceptors are essential for the PFC regulation (68, 69). D1 receptors can regulate the sustained firing of the PFC neurons in a delay period in the delayed response task (70). It is thus generally believed that the optimal PFC functions are likely dependent on NE stimulation of the postsynaptic α2A-adrenoceptors and dopaminergic stimulation of D1 receptors (51, 71). Nevertheless, it remains unclear how DA and NE systems coordinate their action to optimize the PFC functions (32).

Here, we investigated the influence of the α2A-adrenoceptor agonist and antagonist (Guanfacine and Yohimbine, respectively) on spatial memory of DAT-KO rats. It is known that α2A-adrenoceptor agonist GF can improve working memory in aged monkeys and rats and shows therapeutic efficacy in ADHD patients (50). It is believed that GF acts within the PFC *via* postsynaptic α2A-adrenoceptor on the dendritic spines inhibiting cAMP-PKA opening K^+^ channel signaling and causing activation of PFC neurons leading to the improvement of cognitive functions (51). In spontaneously hypertensive rats (SHR), GF caused a decrease in DA release in the prefrontal cortex, but NE concentrations were increased, and thus the NE system appears to be hyperactive as a result of α2A-adrenoceptor activation. These results indicate that GF can improve deficient NE modulation of neuronal circuits in the PFC in this animal model of ADHD (72). At the same time, the blockade of the α2A-adrenoceptors with YOH leads to a deficiency in impulsive decision making in delayed response tasks in rats (56, 73–75). Furthermore, the acute administration of YOH induces ADHD–like behaviors by increasing locomotor hyperactivity and impulsivity of rats (76). Infusion of YOH into the PFC in monkeys impaired working memory control of attention (77).

As with other experimental settings, DAT-KO rats demonstrated pronounced hyperactivity in the maze compared to WT animals. However, their activity level has not been significantly altered during training to the task compared to the pre-training period in wall-less maze. In contrast, WT animals significantly increased the distance traveled during training in comparison with the pre-training period. It is likely that in WT rats, the level of exploratory activity and attention was higher than in DAT-KO rats, and thus, the distances traveled in the maze increased as they were learning the task.

The level of motivation is also essential for task performance. In our study, we compared the preference of reward to regular food in DAT-KO and WT rats. While the reward preference seems to be similar in both groups of rats, the difference in body weight decreases during experimental tasks may suggest that potential minor alterations in the motivation of DAT-KO rats can not be fully excluded. Furthermore, potential contribution of transient anxiety phenotype in DAT-KO rats (38) to the findings observed can not be ruled out.

Despite their prominent hyperactivity, trained drug-naive DAT-KO rats took longer to reach the goal box than their WT counterparts. In addition, WT rats seem to acquire task rules faster than DAT-KO rats, and the drugs used influenced them differently than spontaneously hyperactive DAT-KO rats. It appears likely that due to inattentiveness and the deficient spatial working memory, DAT-KO rats showed poor performance in solving tasks and searching for a correct path to the goal. Furthermore, the DAT-KO rats also demonstrated an increased number of returned runs indicating the perseverative pattern of activity in task solving.

Guanfacine administration had only a minimal inhibitory effect on the distances traveled by DAT-KO rats in the maze. In contrast, YOH administration significantly increased the distance traveled. Likely, the potential inhibitory action of GF on hyperactivity was not revealed to a full extent due to experimental conditions in the maze. Therefore, further studies in locomotor activity monitors are necessary to evaluate the inhibitory action of GF in hyperactive DAT-KO rats and compare it to that of amphetamine and methylphenidate in DAT-KO rats (37) and mice (61).

Both GF and YOH did not affect the time necessary to reach the goal box compared to untreated DAT-KO rats but exerted opposite effects on the number of abnormal return runs, reflecting a perseverative pattern of locomotor activity of mutants. It is well known that hyperactivity in DAT-KO rats is often accompanied by stereotypical (perseverative) patterns of activity in various settings (38, 41, 43). In hyperdopaminergic DAT-KO rats, GF decreased, and YOH increased this type of activity, indicating that GF may optimize the PFC functions and thus diminish the ADHD-like behavior while YOH is aggravating it.

Similar opposite effects of these treatments were observed when the time spent in the maze error zones was analyzed. The DAT-KO rats are known to have deficient working memory (43). The mutants indeed spent more time in the error zones in the Hebb-Williams maze time, indicating an impaired level of task acquisition. Administration of GF ameliorated this deficit, while YOH exacerbated it. These data strongly indicate that GF can improve the consolidation of spatial information under the condition of hyperdopaminergia and consequently can ameliorate their spatial memory deficit. The role played by the noradrenergic modulation in memory consolidation processes was described in numerous investigations (57, 78, 79).

The effects of GF in WT rats were not so conclusive due to the lack of hyperdopaminergia to reveal its beneficial actions. At the same time, YOH caused locomotor activation, increased the duration of task fulfillment, time spent in the error zones of the maze, and the number of visits to the error zones in WT rats indicating impaired cognitive function following α2A-adrenoceptor blockade. These data support previous observations showing that YOH at low doses (0.5–2 mg/kg, i.p.) can induce locomotor activation in rats (73, 80) and increase impulsivity in the five-choice serial reaction time task (74). It was also shown that Guanfacine induces activation in the PFC leading to the decline of hyperactivity in DAT-KO rats, whereas Yohimbine may provoke hyperactivity by increasing dopamine synthesis and intraneuronal dopamine stores (81).

Our experiments found the opposite effects of agonist and antagonist of α2A-adrenoceptors on the spatial working memory of hyperdopaminergic DAT-KO rats. This might result from different influences exerted by the PFC on the subcortical structures *via* the noradrenergic and dopaminergic systems. In addition to working memory, Guanfacine, the agonist of α2A-adrenoceptor improves many PFC functions (51). It has been suggested that in SHR rats, abnormal behaviors may result from a disbalance between increased noradrenergic and decreased dopaminergic systems regulation in PFC (69). The complementary role of DA and NE in the PFC and their interactions for memory facilitation was discussed by other investigators (32, 67). It was proposed that beneficial effects of GF arise *via* strengthening PFC network connectivity as a consequence of NE actions on postsynaptic α2A-adrenoceptors dendrite spines in PFC (51, 82).

## Conclusion

These results indicate that Guanfacine may positively affect ADHD-like abnormal behaviors of DAT-KO rats. This drug improves spatial memory deficit in DAT-KO rats, thus likely contributing to attention improvement. The treatment of DAT-KO rats with Yohimbine, an antagonist of α2A-adrenoceptors, exacerbates ADHD-like behaviors. These data further support the translational value of DAT-KO animals as a useful animal model to investigate pathological processes and discover new ADHD treatments.

## Data Availability Statement

The raw data supporting the conclusions of this article will be made available by the authors, without undue reservation.

## Ethics Statement

The animal study was reviewed and approved by the Ethics Committee of Saint Petersburg State University, St. Petersburg, Russia.

## Author Contributions

NK, AV, and RG designed the study. NK, AB, and AG performed the experiments and data analysis. NK, AI, and RG writing and editing of the manuscript. RG and AV contributed to the conceptualization and funding acquisition for the project. All authors contributed to the article and approved the submitted version.

## Conflict of Interest

The authors declare that the research was conducted in the absence of any commercial or financial relationships that could be construed as a potential conflict of interest.

## Publisher’s Note

All claims expressed in this article are solely those of the authors and do not necessarily represent those of their affiliated organizations, or those of the publisher, the editors and the reviewers. Any product that may be evaluated in this article, or claim that may be made by its manufacturer, is not guaranteed or endorsed by the publisher.
